# Myasthenic crisis appearing after resection of intracardiac ectopic thymoma with superior vena cava syndrome

**DOI:** 10.1186/s40792-021-01233-4

**Published:** 2021-06-15

**Authors:** Satoshi Takebayashi, Yasuji Yoshikawa, Masato Morita, Ryotaro Nagashima, Yuichi Nakazono, Shinji Miyamoto

**Affiliations:** 1 The Department of Cardiovascular Surgery, National Hospital Organization Beppu Medical Center, 1473 Uchikamado, Beppu, Oita 874-0011 Japan; 2 The Department of Pathology, National Hospital Organization Beppu Medical Center, 1473 Uchikamado, Beppu, Oita 874-0011 Japan; 3 The Department of Cardiovascular Surgery, Oita University Hospital, 1-1 Idaigaoka, Hasama-machi, Yufu, Oita 879-5593 Japan

**Keywords:** Ectopic thymoma, Superior vena cava syndrome, Myasthenia gravis, Myasthenic crisis

## Abstract

**Background:**

We describe herein an extremely rare case of intracardiac ectopic thymoma—only two pure cases have been reported to date—associated with myasthenia gravis, an infrequent complication of ectopic thymoma.

**Case presentation:**

A 71-year-old woman with superior vena cava syndrome was found to have a large mass mainly located in the right atrium. Tumor resection under cardiopulmonary bypass was performed. The pathological diagnosis was type AB ectopic thymoma. The postoperative course was complicated by progressive respiratory failure, and she was diagnosed with myasthenic crisis based on clinical signs and the edrophonium test. The patient recovered and was weaned from prolonged mechanical ventilation after receiving intravenous immunoglobulin, and was subsequently discharged uneventfully.

**Conclusions:**

This is the first report of myasthenic crisis due to intracardiac ectopic thymoma. Residual thymoma is a risk factor for the development of post-thymectomy myasthenia gravis, and long-term follow-up is required.

## Background

Ectopic thymoma is a rare neoplasm accounting for 4% of all thymomas, arising mostly in the cervical region, lungs, pleura, thyroid, pericardium, and middle/posterior mediastinum [1]. Intracardiac ectopic thymomas are extremely rare, with only four cases (including two pure tumors) reported to date in the English literature [2–4]. Ectopic thymomas are only sporadically linked with myasthenia gravis (MG) [5]. We describe herein an extremely rare case of ectopic thymoma of the right atrium (RA) with superior vena cava (SVC) syndrome and post-thymectomy myasthenic crisis (MC).

## Case presentation

**Fig. 1:**
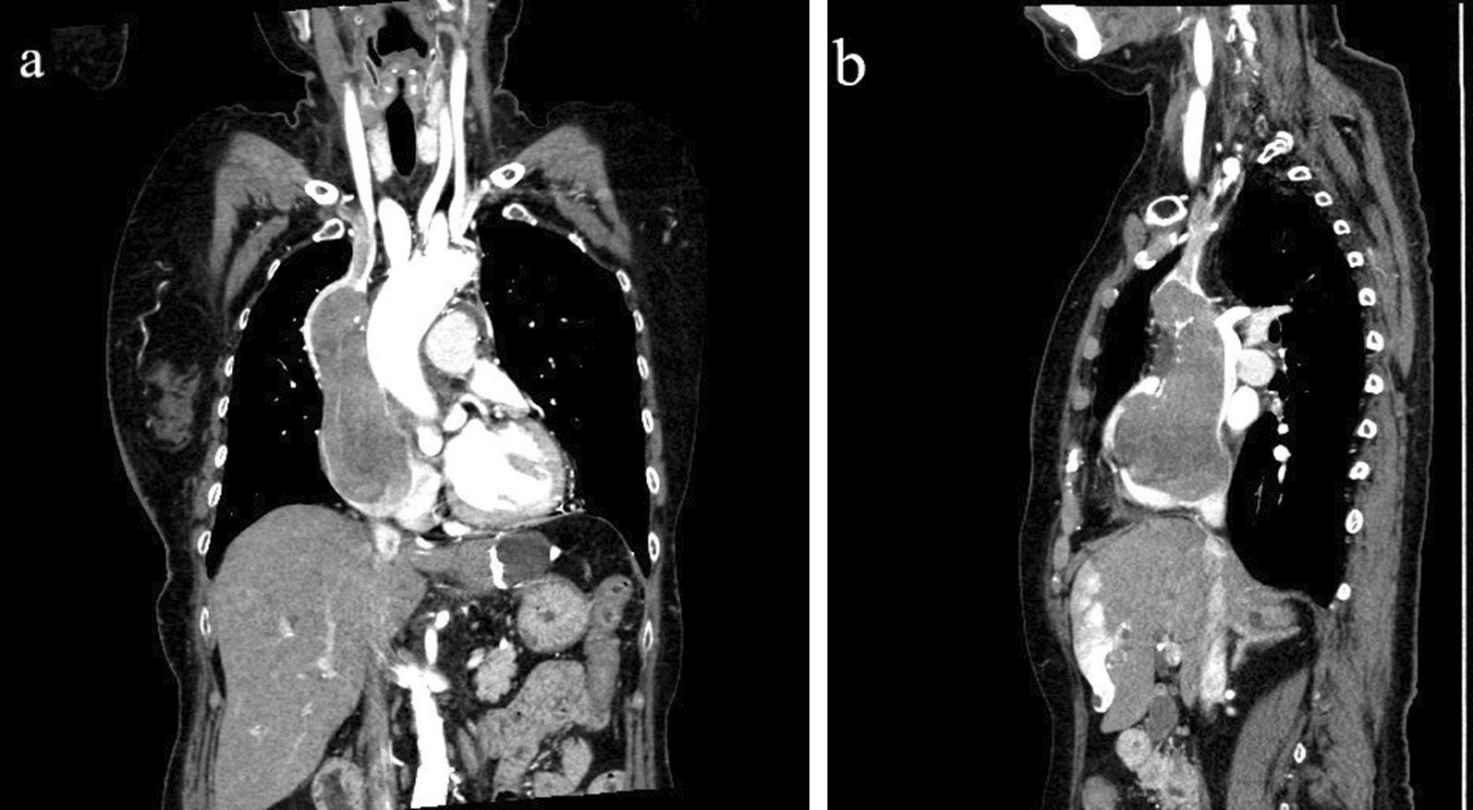
Preoperative contrast-enhanced computed tomography shows a large mass in the right atrium extending to the superior vena cava and left brachiocephalic vein. **a** Coronal view; **b** sagittal view

**Fig. 2:**
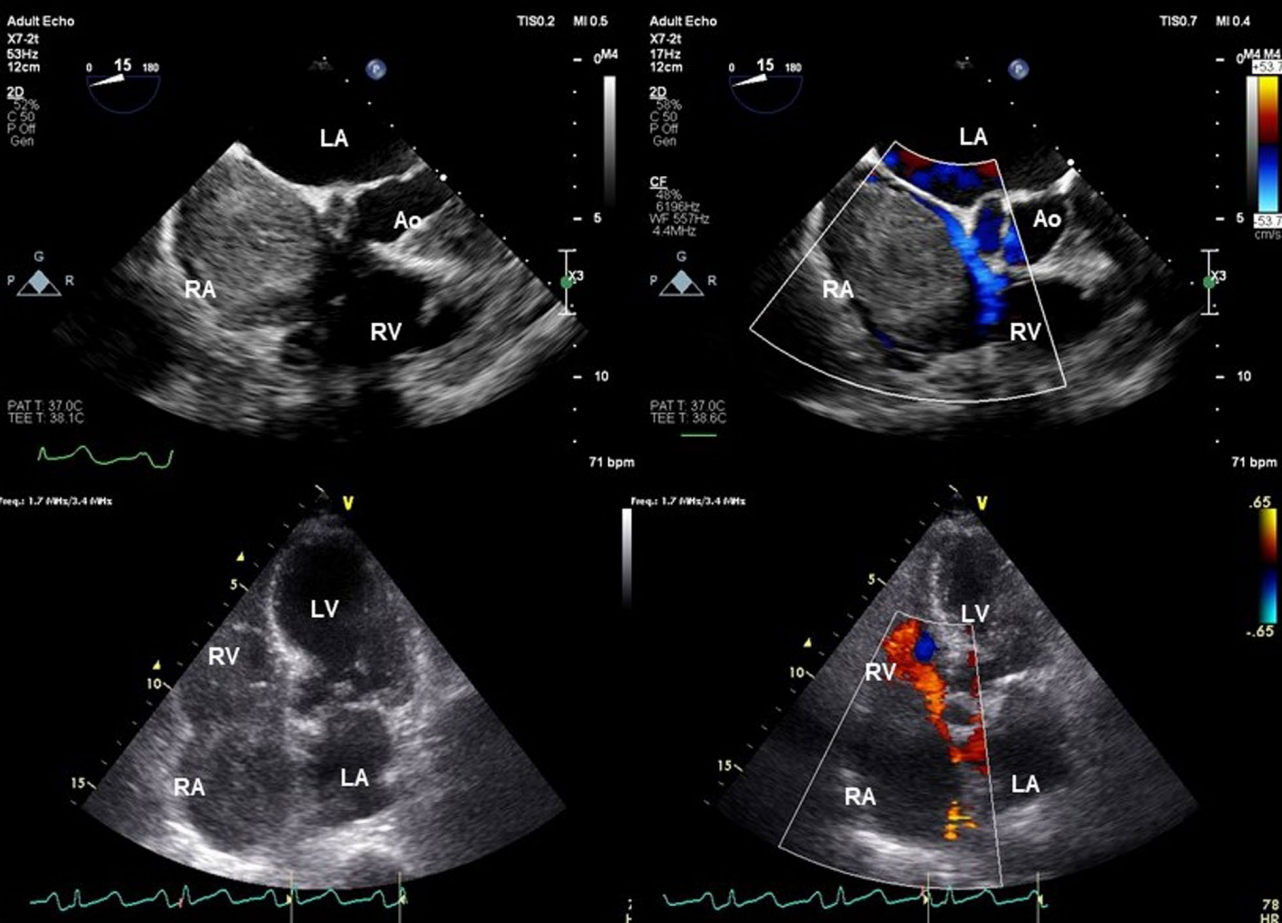
Transthoracic and transesophageal cardiac ultrasonography demonstrate a large mass nearly filling the right atrium. *RA* right atrium, *RV* right ventricle, *LA* left atrium, *LV* left ventricle, *Ao* aorta

**Fig. 3:**
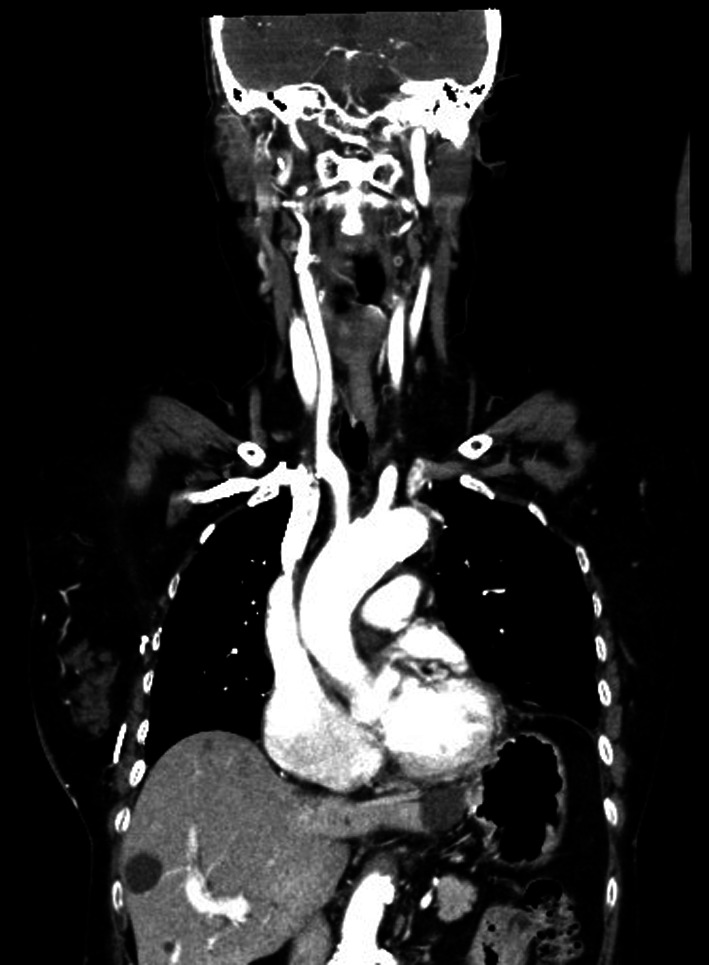
Postoperative contrast-enhanced computed tomography shows no mass in the superior vena cava or right atrium

A 71-year-old East Asian woman with a 6-month history of right upper extremity swelling was referred to our hospital. She had undergone resections of a thyroid tumor at 35 years old and brain tumor at 65 years old, neither of which had shown any sign of recurrence. She showed no sign of MG preoperatively. Results of blood tests and electrocardiography were unremarkable. Preoperative vital capacity was 84.3% and forced expiratory volume in 1 s was 66.67%. Computed tomography (CT) revealed a large mass with a diameter of around 12 cm occupying the RA and extending into the SVC and left brachiocephalic vein (LBCV) (Fig. 1). No lymphadenopathy was detected on CT. Transthoracic and transesophageal cardiac ultrasounds revealed a partially mobile, 5.6 × 5.4 cm mass adherent to the anterior side of the right atrial wall (Fig. 2). To avoid tumor embolization and resolve the SVC syndrome, resection of the intracardiac tumor was performed via median sternotomy. The absence of any mass in the mediastinum was also confirmed during the surgery. A patch of pericardium was harvested in advance for the RA and SVC reconstructions. Cardiopulmonary bypass (CPB) was instituted after cannulation of the inferior vena cava through the right femoral vein and the ascending aorta. The RA was opened under induced ventricular fibrillation, revealing a large mass. The tumor was firmly adherent to the anterior RA and SVC walls and the LBCV. The tumor was removed en bloc with the involved wall of the RA, SVC and LBCV. The SVC and RA wall defect were repaired with an autologous pericardium patch. The LBCV was bypassed by anastomosing an 8-mm conduit (Gore Propaten vascular graft; W. L. Gore and Associates, Flagstaff, AZ, USA) to the RA wall in an end-to-side manner. The patient was weaned off the CPB uneventfully, and was extubated uneventfully on postoperative day 1. However, she started to complain of severe dyspnea on postoperative day 7 and required mechanical ventilation for respiratory deterioration with atelectasis of bilateral lower lobes and mild pneumonia from postoperative day 9. Improvement of collapsed bilateral lower lobes led to extubation on postoperative day 14. However, she complained of dyspnea again, 10 min after extubation due to carbon dioxide (CO_2_) narcosis with a PaCO_2_ of 66.7 mmHg and a PaO_2_ of 54.6 mmHg. The patient was immediately reintubated and again required mechanical ventilation support. She complained of dyspnea again 4 h after another attempt at weaning from ventilation on postoperative day 18, and histopathological diagnosis of the resected mass identified ectopic thymoma. The possibility of myasthenic crisis (MC) was therefore considered despite negative results for anti-acetylcholine receptor (AChR) antibody and anti-muscle-specific tyrosine kinase (MuSK) antibody. An edrophonium test showed temporary improvement of tidal volume (from 350 mL/min to 400 mL/min) and grip strength (from 10/13 kg to 13/17 kg, right/left, respectively) on postoperative day 19 resulted in a diagnosis of MC. Intravenous immunoglobulin (IVIg) was initiated at 20 g/day for 5 days from that day. She was extubated on postoperative day 32 after confirming a negative response to the edrophonium test. Postoperative CT revealed no masses in the SVC or RA (Fig. 3). Swelling of the right upper extremity improved and she was discharged uneventfully at 25 days after the last extubation.

**Fig. 4:**
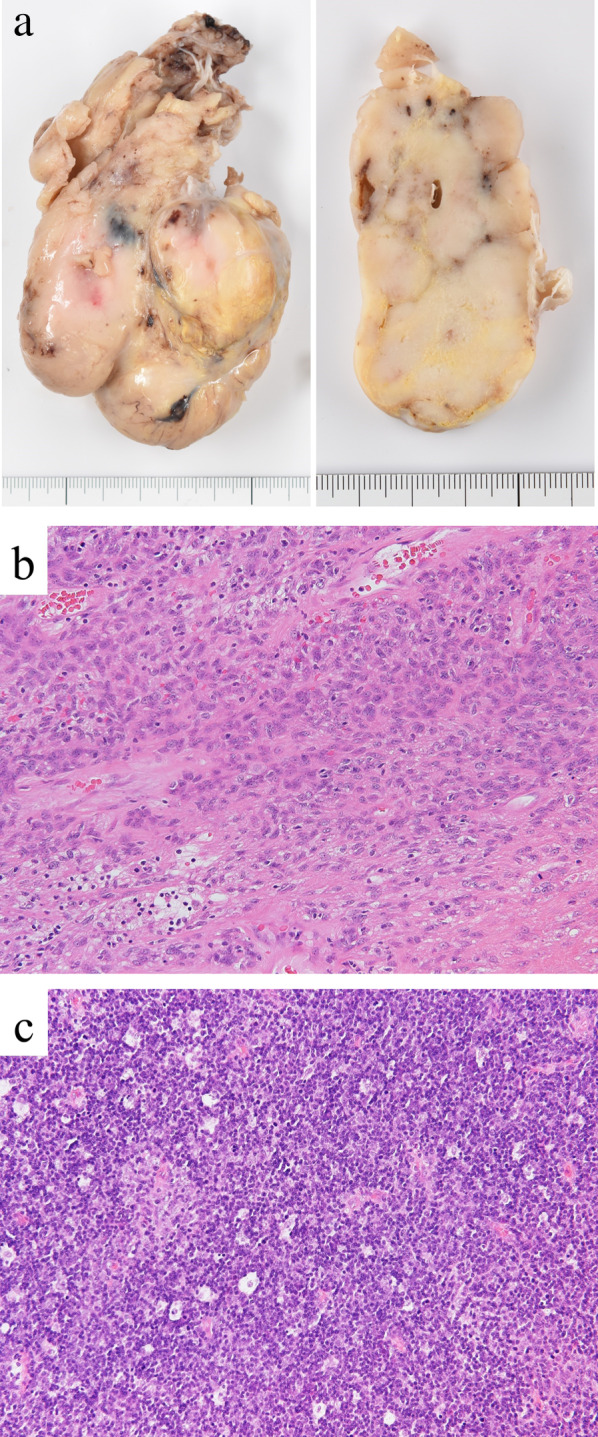
Pathology of the resected tumor. **a** Gross image of the intracardiac mass showing a bosselated outer appearing light tan in color (left) and a lobulated cut surface with small cysts (right). Note a completely smooth surface of the tumor. **b** A representative histological image of a lymphocyte-poor (type A-like) area. Tumor cells with oval, bland nuclei and eosinophilic cytoplasm proliferate in solid sheets. Small numbers of lymphocytes are seen (hematoxylin–eosin stain, × 200). **c** Histological image of a lymphocyte-rich (type B-like) area. A small number of epithelioid tumor cells are intermingled along with a starry sky appearance (hematoxylin–eosin stain, × 200)

The excised specimen showed a bosselated mass measuring 85 × 70 × 35 mm, elastic hard in consistency with a smooth surface (Fig. 4a). The cut surface revealed a solid mass, pale tan in color and with lobulation. Histologically, the tumor showed two components: portions of lymphocyte-poor epithelioid or spindle cell area (Fig. 4b), and portions rich in lymphocytes with scattered epithelioid cells (Fig. 4c). The epithelial cells were spindle-shaped, oval, or polygonal without atypia. Immunohistochemically, TdT-positive immature T cells were abundant in the lymphocyte-rich areas. The tumor was completely covered by a fibrous capsule without tumor cell invasion. The pathological diagnosis was type AB thymoma.

## Discussion

Thymomas are epithelial neoplasms of the thymic gland, with 96% arising in the anterior/superior mediastinum. The remaining are ectopic thymomas that mainly arise from ectopically dispersed thymic tissue, mostly in the neck, lungs, pleura, thyroid, pericardium, and middle/posterior mediastinum [1]. The histogenesis of ectopic thymomas is widely considered to involve thymic tissue displaced during embryonic development. Embryologically, the thymus originates from the third and to a certain extent fourth pharyngeal sacs. During the eighth gestational week, the thymic primordia gradually migrates toward the anterosuperior mediastinum. One hypothesis for the origin of ectopic thymomas is that neoplasia arises from thymic tissue misplaced during the embryonic descent. However, the cardiac system develops during the third gestational week, earlier than the development of the thymus. This hypothesis thus cannot explain thymomas arising in cardiac tissue. Another hypothesis is that ectopic thymomas can derive from stem cells that have retained the ability to differentiate into various cell lineages. These two are the theories for the origin of ectopic thymomas that have been most widely accepted [1, 3].

The thymoma in our patient occupied the RA, SVC and LBCV, and had not spread outside the heart and vessels. The main mass was located in the RA lumen almost freely from which tumor branches extended in the lumen of SVC and LBCV. Because the original thymus had involuted into a small, solid tissue, the intracardiac thymoma was reasonably thought to have originated from the RA. To the best of our knowledge, four cases of intracardiac ectopic thymoma have been reported in the English literature [2–4]. However, two of them arose within cardiac myxomas [4], so only two cases of pure intracardiac thymoma have been reported [2, 3]. Ectopic thymomas are only sporadically linked with MG [5]. This is the first report of MC associated with pure intracardiac ectopic thymoma.

Anti-AChR or anti-MuSK antibodies are found in over 80% of patients with MG. Cases of MG without detectable antibodies against AChR or MuSK are seen in around 15%, and are termed “double-seronegative MG” [6]. In our patient, despite double-seronegativity, MC was able to be diagnosed according to the clinical signs and a positive result from the classic edrophonium test. The patient actually recovered and was weaned from prolonged mechanical ventilation after IVIg administration. The main therapies for MC are IVIg and plasma exchange (PE) [7]. IVIg therapy may offer similar efficacy to PE, but with fewer complications. The effect of those therapies lasts for only a few weeks, so corticosteroids are often used concurrently with IVIg and PE [8, 9]. IVIg was simply used for treatment in this case without administration of corticosteroids, as she showed MC associated with mild pneumonia.

Approximately one-third of patients with thymoma display MG [10]. Thymectomy plays a role as a primary treatment in thymoma patients with MG, who usually show excellent prognosis [11]. Nevertheless, MG recurs in some patients with thymoma after thymectomy, and such post-thymectomy MG is attributed to high serum titers of auto-antibodies against AChR [11–13]. Our patient developed MG post-thymectomy despite no history or symptoms of MG pre-thymectomy and was negative for both AChR and MuSK antibodies post-thymectomy. Some studies have suggested that thymomas can actively export large numbers of mature autoantigen-specific T cells into the peripheral blood, and those cells can persist in stimulating autoantibody production [14]. In our patient, a comparison of the number of T cells between pre- and post-thymectomy was not available. In addition, the test for low-density lipoprotein receptor-related protein 4 (LRP4) antibodies, which have been identified as a potential autoantibody target in several double-seronegative MG patients, was not performed on our patient. However, LRP4-positive patients tend to be < 50 years old and predominantly show ocular or mild disease [9], suggesting that this form may have been unlikely in our patient. The reason for the late onset of post-thymectomy MG remains unclear. Very subtle symptoms of MG that were overlooked preoperatively might be one possible explanation. Predicting the natural course of ectopic thymoma is difficult due to the low incidence of the disease. Therefore, the optimal treatment of patients with ectopic thymomas is currently considered to be complete surgical resection. Incomplete resection represents a risk factor for the development of the post-thymectomy MG [13]. Recurrence of intracardiac invasive thymoma has been reported [15]. We resected the intracardiac thymoma in the present patient radically and confirmed the absence of masses in the heart and vessels on postoperative CT. As of the time of writing, the patient has remained free of symptoms for more than 9 months since surgery. Long-term follow-up is required, given the possibility of late recurrence [15].

## Conclusions

We herein report a case of MC due to intracardiac ectopic thymoma. The tumor was resected radically and the patient has remained symptom-free. Residual thymoma is a risk factor for the development of post-thymectomy MG, and long-term follow-up is required in our patient.

## Data Availability

Please contact author for data requests.

## Abbreviations

CT: Computed tomography
SVC: Superior vena cava
LBCV: Left brachiocephalic vein
CPB: Cardiopulmonary bypass
MG: Myasthenia gravis
MC: Myasthenic crisis
AChR: Acetylcholine receptor
MuSK: Muscle-specific tyrosine kinase
IVIg: Intravenous immunoglobulin
PE: Plasma exchange
RA: Right atrium
RV: Right ventricle
LA: Left atrium
LV: Left ventricle
Ao: Aorta
